# Professional Perspectives on Children’s Health Assets: A Delphi Study

**DOI:** 10.3390/healthcare12050506

**Published:** 2024-02-20

**Authors:** María Isabel Vidal-Sánchez, Pablo A. Cantero-Garlito, Ángel Gasch-Gallén

**Affiliations:** 1 Physiatrist and Nursing Department, Health Science Faculty, Zaragoza University, 50009 Zaragoza, Spain; vidal@unizar.es (M.I.V.-S.); angelgasch@unizar.es (Á.G.-G.); 2 GIIS104-Wellbeing, Occupation, Participation and Health Research Group (IBiOPS), Institute for Health Research Aragón, Zaragoza University, 50009 Zaragoza, Spain; 3 Nursing, Physiotherapy and Occupational Therapy Department, Health Science Faculty, Castilla La Mancha University, 45600 Talavera de la Reina, Spain; 4 GIIS094-Research Group Nursing Research in Primary Care in Aragón (GENIAPA), Institute for Health Research Aragón, Zaragoza University, 50009 Zaragoza, Spain; 5 GIIS011-Aragonese Research Group in Primary Care (GAIAP), Institute for Health Research Aragón, Zaragoza University, 50009 Zaragoza, Spain

**Keywords:** childhood, health assets, health promotion, community health, occupational therapy, Delphi

## Abstract

This study aims to describe a local community expert’s perspective on the identification of and access to children’s health assets and to gather proposals to promote children’s health and well-being within their community. The health asset approach is essential for health promotion, and there is evidence of its benefits to individuals’ or communities’ health when this approach is observed. Children’s health assets are gaining increasing interest, but the literature that captures the perception of professionals working with children is scarce. Qualitative research designed with Delphi methodology was carried out with the participation of 25 professionals working in a neighbourhood with children and families. The participants stated that this neighbourhood was a good environment for the healthy and happy growth of children but pointed out that there were inequities. They emphasised the importance of economic and physical security and feeling loved. The absolute best aspects of the neighbourhood according to these experts were its support networks, mutual help, educational and health services, and green spaces, and the most deficient aspects were the possibility of a hopeful future and emotional support within the family unit. Poverty and/or the scarcity of economic resources were identified as the main barriers to accessing health assets. Special difficulties in access to health for migrant and Roma children were also identified. The panel of experts made concrete action proposals. It was recommended to support resources and services that already exist in their community. The experts prioritised work with families, education, working in conjunction with vulnerable groups, community participation, and networking.

## 1. Introduction

Health promotion understands health as an intrinsic and positive force [1]. One of the most solid bases for health promotion from this perspective is Antonovsky’s salutogenic theory [2]. This theory focuses on answering these questions: What creates health? What causes individuals and communities to improve and control their health? [2,3,4]. Salutogenic theory can be seen as the theoretical basis for health-asset-based approaches [3]. In the growing literature on health assets, there are various definitions, but the most widespread is still the one coined by Morgan and Ziglio in 2007 as “any factor (or resource) that enhances the capacity of individuals, groups, communities, populations, social systems and institutions to maintain and sustain health and well-being, and helps them to reduce health inequalities” [5]. Therefore, the health asset (HA) model [6] is essential for the promotion of health associated with a more positive, holistic, and complex vision, as a fundamental human right [7,8]. This approach promotes the agency capability of individuals and communities in the management and control of their health [6,9,10]. It focuses on developing the strengths and capabilities of communities to promote their well-being and take control of their health, which requires their full involvement [11].

One of the fields in which evidence of the benefits of HAs has been collected is research on HA recommendation, also called social prescribing (although there are differences in the inherent views in each one) [9]. HA recommendation is largely determined by local contexts [12]. Some notable outcomes of using this strategy that have been reported are improvements in emotional well-being, physical health, lifestyle, social networking, motivation, optimism about the future and learning and acquiring new interests; reduced isolation and loneliness; increased self-esteem; empowerment; satisfaction; sociability and communication skills; and the more positive use of health services [13].

There is an association between social prescription (HA recommendation) and occupational therapy, as this discipline has a long history of recommending activities to improve the health and well-being of individuals, groups, and communities [14]. Therefore, in this paper, special emphasis is placed on occupations as understood by occupational therapy.

HAs should, by definition [5], help to reduce health inequities. The social determinants of health (SDHs), according to the World Health Organisation’s Global Commission on Social Determinants of Health [15], are “the structural determinants and conditions of everyday life” that are “responsible for a significant part of health inequities between and within countries” [16,17]. In the CSDH framework [18], a distinction is made between “context”, “structural stratifiers”, and “intermediate determinants of health”. Together, these are the main social causes that underlie multiple health outcomes through complex and diverse pathways [19]. The inclusion of social determinants in research is often accomplished by incorporating gender, origin, ethnicity, class, and race (among others). However, it is important not to overlook the biases that can result from using these sociodemographic indicators as explanatory variables without considering the underlying social processes, such as institutional discrimination [20].

CSDH [15] identifies early development as a key part of the pathways and mechanisms through which social inequities in health are generated and perpetuated in adulthood. For this reason, this study focused on childhood.

In order to introduce the social determinants that emerged in this research, it is necessary to contextualise them, following the recommendations for health promotion research [3,7]. This study was carried out in the neighbourhood of Torrero-La Paz in Zaragoza, Spain. Torrero-La Paz is a working-class neighbourhood that grew from 1950 onwards with the arrival of Roma families and families who emigrated from other parts of Spain, especially Andalusia. Today, the neighbourhood is home to families of very heterogeneous socioeconomic levels, cultures, and origins. The Roma people constitute the main ethnic minority in the neighbourhood, and migrants account for 16% of the total population. At the urban planning level, there are also great inequities between newly built and old houses and streets.

In this context, and with this population group (children), we took into consideration, among others, gender, origin and migratory status, ethnicity, racialisation, and socioeconomic status. Gender is one of the main axes of health inequities [21,22,23]. Origin and migratory status are associated with health inequities, even in later generations [24]. Regarding ethnicity, in Europe, the Roma population is the largest ethnic minority and suffers from a clear situation of inequality in health, both in perceived health and in lifestyles and access to certain health services. They are also the sector of society that perceives the most discriminatory practices in their daily lives. Arza and Rodríguez [25] identified two professional models towards intercultural competence with the Roma population: an openness model and a blocking model. As for racialisation, it can be defined as a social construct that persists as a strong axis of inequity in the collective imaginary [26]. Focusing on the Afro-descendant population in Spain, Rodríguez-García et al. [26] stated that it causes the stigmatisation of racialised people, which extends generation after generation, even within mixed (Spanish African) families. There is evidence that social and economic disadvantages are associated with poorer outcomes on health indicators and lower life expectancy [16,27], and social gradients in health have been evidenced for most child health problems [28].

Childhood and youth are crucial stages in laying the foundations for healthy development [29]; however, there are few studies that focus on social inequities in children’s health, fewer still in Spain [23].

Regarding publications on child and youth HAs, the perceptions of adolescents, children, and their families have been recorded. Internal and external HAs were identified in these investigations [6,30,31,32]. Focusing on HAs for children under 13 years of age, Whiting et al. [31] identified one stabilising asset (“being a family”) and eleven core assets (internal and external) in their study with participants aged 9–11 years. The external HAs they identified were friendships, personal belongings, and community (referring to their neighbourhood). The internal assets they identified were “pride”, “having fun”, “self-identity”, “being physically active”, “resourcefulness”, “I’m growing up”, “engagement” and “self-worth”. Eriksson et al. [33], showed that the places where children live, grow, and play have a strong influence on their health and well-being and incorporated the concept of social capital to deepen the understanding of the interrelationship and interaction between material and social factors in a community. Another study was carried out in the same community this research focused on, with the participation of 130 children (70 boys and 60 girls) and 58 families [30]. One of its main findings was that well-being was linked to occupations in 80% of children and 84.5% of family participants, highlighting the importance of “belonging” and its particular relevance to occupation [34,35] and contributing to the understanding of the interrelationship and interaction of HAs of “doing” with the so-called “being” and “having” HAs, which in turn connect to external (community-level) assets [6]. Another finding of this study was that families felt it was a good neighbourhood for children, with differences according to their origin, and the main determinants of healthy and happy growth identified were physical safety (feeling safe), being with family, and having people who love them very much and access to healthcare [30]. For children, the most health-related places were in nature, and families identified the following as external HAs of the neighbourhood: parks, civic centre, library, and healthcare centres [30]. This study also reported the influence of gender, ethnicity, origin, and other social determinants on the identification and valuation of and access to childhood assets in a local community [30].

While previous studies have primarily focused on the perceptions of children and families, there remains a very limited amount of research on the perceptions of practitioners, within the framework for children’s health asset approach, contextualised in a specific community. Previous research on professionals’ perceptions of HAs contextualised in a local community does not focus specifically on children [36,37]. Their findings point to the identification of healthy physical environments (especially green spaces) and health and social services as assets [36]. In addition, these previous studies recognise socioeconomic and cultural factors as facilitators and barriers to accessing HAs and highlight poverty and lack of education [36,37], adding language barriers and acculturation from people outside their dominant culture [37]. Lack of services is one of the main obstacles to accessing health. Assets and health education; improved health policies; and social, health, and educational services that should be culturally competent are recommended [36,37]. Previous research on local professionals’ perceptions of HAs has some limitations: There are no studies that focus on children’s HAs or include a gender perspective. These studies were conducted in low socioeconomic neighbourhoods with homogeneity in socioeconomic status, which did not allow for a comparison of access to HAs among people in different statuses, and their publication date is quite old (2017 and 2009). However, previous research on local professionals’ perceptions of HAs also has strengths, including being contextualised in a local community and proposing strategies to promote HAs.

In addition, no research has been performed in Spain on professionals’ perceptions of children’s HAs, contextualised in a specific community. Although there are some studies on HAs in local communities that included professionals in social and health sectors among their participants [38].

Therefore, there is a gap in research on professionals’ perceptions of children’s HAs. This study focused on children’s HAs in a particular neighbourhood in a city in Spain, and its main objective was to describe the perspectives of experts on (1) the identification of and access to health assets for children and (2) proposals for promoting children’s health and well-being in this community.

## 2. Materials and Methods

### 2.1. Type of Study

This study is a qualitative design with Delphi methodology. The Delphi methodology is a structured method for systematically collecting expert opinions on a problem, processing the information, and reaching a general agreement as a group. It offers a flexible approach to gathering expert opinions on an area of interest, and it also allows an element of reflection that is lacking in studies based on individual interviews or focus groups. This methodology facilitates the transformation of the individual judgements of experts during research into a higher collective judgement [39,40]. The use of this tool is recommended in cases where there is scarce empirical evidence, subjective factors predominate, and the data are vague [41]. In order to conduct a Delphi study properly, the following principles should be observed: (1) It consists of an iterative process: Successive rounds of consultations must be carried out to give participants the opportunity to review their opinions. (2) It requires feedback: Experts receive the opinions of all participants before the next round. (3) There must be anonymity for individual responses to avoid the effect that an opinion leader can have. (4) It aims to build consensus [39].

### 2.2. Context

This study was carried out in the neighbourhood of Torrero-La Paz in Zaragoza, a city of 666,880 inhabitants, located in the Northeast of Spain. The neighbourhood is one of the green lungs of the urban area of Zaragoza, as it is home to the Pinares de Venecia (area of pine fields) and the Imperial Canal. It is currently one of the city’s districts that is best served by public transport. This neighbourhood is known for having a fighting and vindictive personality, and it is recognised in the city as one of those with the greatest drive by its people, due to its high number of activities, projects, and associations in proportion to its size.

### 2.3. Participants

This research focused on collecting the opinions of experts in child health and well-being in this community. The term expert is ambiguous, so we used the definition of García and Suárez (2013), who defined experts as people with prior training or experience that gives them a mastery of a subject that is superior to the average level of their peers. Expert opinions can be used as conclusive judgements [39]. Study participants were selected for their professional experience with children and/or their families in the Torrero-La Paz neighbourhood. Professional experience and expertise were understood as having worked in the neighbourhood in which the study was carried out for a minimum of one and a half years and having been recommended as an expert on the subject by key agents in the community.

To ensure the comprehensive and fully representative coverage of all viewpoints, professionals from all sectors, as well as the various fields and professions within them, were contacted: social (social work, social street education, social education in resources for children in vulnerable situations, psychology, and intercultural mediation); healthcare (paediatrics, nursing, and general medicine); formal education (therapeutic pedagogy, primary education, and infant education (teachers and teaching assistants); and non-formal education (leisure and free time). In addition, this study sought to ensure that the participants were representative of professionals working exclusively with children, exclusively with families, and with both.

The following steps were taken to identify experts for participation in the panel of experts:

- Identifying resources, services, and professionals working with children;
- Identifying resources known and used by children and their families in the neighbourhood;
- Contacting by telephone and conducting face-to-face interviews with key agents in the community;
- Compiling contacts of possible participants.

Based on these data, through convenience sampling, a large group of experts was preselected in anticipation of possible losses during the study, contacting 40 professionals from formal and non-formal education, health, and social services who had to meet the following inclusion criteria:

- Have a minimum of one and a half years of experience in working directly with children and families in the neighbourhood;
- Having been recommended as an expert on the subject by key agents in the community.

**Figure 1:**
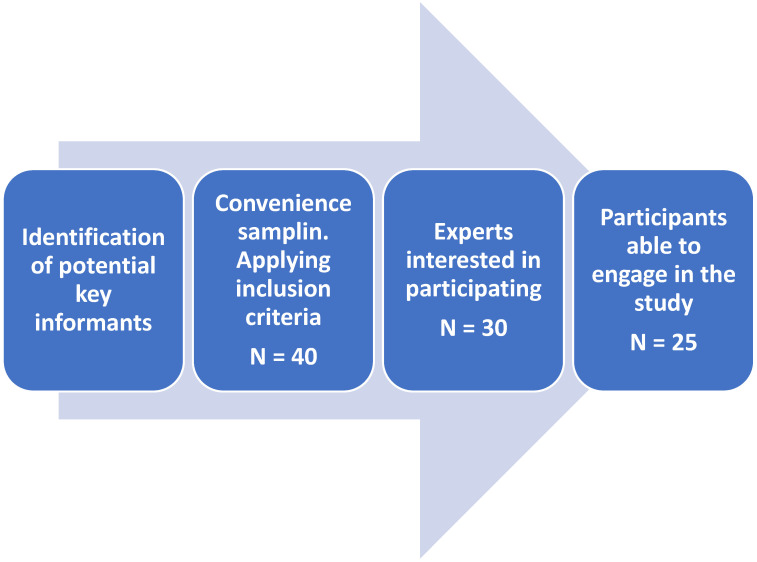
Expert panel participant selection process.

After establishing contact with the 40 potential key informants and sending two letters of presentation and information about the study, 30 of the experts contacted agreed to collaborate in this study. After learning about the timeframe and commitment required for their participation, 25 experts participated (see Figure 1 and Table 1).

As can be seen in the table, the 25 participants worked in the social, educational, and health fields. They included 5 primary school teachers, 2 infant school teachers, 1 infant classroom assistant, 1 therapeutic pedagogy teacher, 3 leisure and free time monitors, 2 social educators, 2 street educators, 2 intercultural mediators, 1 psychologist, 1 social worker, 3 general practitioners, 1 paediatrician, and 1 nurse.

### 2.4. Materials

Three in-depth interviews were conducted, one for each area of intervention: social, health, and educational. The results of these interviews were used as the basis for the Delphi study, which was conducted with the aim of seeking consensus from professionals from various fields about their opinions on the identification of and access to health assets for children in the neighbourhood and the proposal and prioritisation of improvement actions to be implemented in that particular context.

In-depth interviews: a semi-structured interview was designed, following the existing recommendations for this method [42], which was sent to three professionals from the areas of intervention with children and families identified in the neighbourhood (health, social and educational) and served as one of the bases for the selection of the questions for Delphi participants. The interview script included questions on age, sex, profession, links with the neighbourhood, years of experience in this community, and interest in the subject, as well as open questions on the concept of health; the state of health of children in the neighbourhood; resources, factors, and characteristics of the neighbourhood that benefit children’s health and what is lacking; opinion on the extent to which some resources and factors, given by the interviewer, improve children’s health; and proposals for action to improve children’s health in the neighbourhood, prioritisation of these, agents and networks necessary to carry them out, and the key actors or experts in children’s health and well-being in this neighbourhood (see Script S1 in Supplementary Materials).

Questions for Delphi participants: The questions were designed ad hoc for this research based on specialised bibliography and the results of the in-depth interviews. In the first round, sociodemographic data of the participants (sex, area of intervention, profession, years of experience in the neighbourhood, population/s with whom they worked in the last 5 years, and whether they live in the neighbourhood) were collected, together with professional perspectives on assets; access to assets related to children’s health and well-being (barriers and facilitators), including a specific section on occupations; and proposals for improvement (see Round 1 Form S1 in Supplementary Materials).

### 2.5. Procedure

The in-depth interviews, lasting approximately one hour and fifteen minutes, were conducted in 2020, and the results were used, together with the specialised literature, to design the Delphi study, contextualising it in the local community of the study.

The Delphi study questions were administered in two rounds through the University of Zaragoza’s Google Forms web tool.

The information was sent via e-mail, and the telephone number and e-mail address of the research team were made available to the participants for any request.

The Delphi study was conducted using a multi-stage iterative process, with each round based on the previous results obtained. The response information from each participant was stored in the database created for the study after receipt by e-mail.

To respond to the first round, in May–June 2021, a deadline of four weeks was established, with a weekly reminder via e-mail and/or telephone.

The research team, after analysing the responses, prepared the questions for the second round, with the aim of reaching a consensus among participants. In the second round, participants were questioned on 12 topics (see Round 2 Form S2 in Supplementary Materials) and a deadline of three weeks for response was established, in September–October 2021, with a weekly reminder via e-mail and/or telephone.

The responses collected in the database were summarised to identify common patterns and trends. The discourse analysis was carried out according to the following sequence: Once the units were established, an open coding process was carried out, following a constant comparison procedure. Then, through an axial coding process, the subcategories were integrated into broader categories. Finally, the categories were grouped into three themes corresponding to the objectives of the study.

## 3. Results and Discussion

The aim of this research was to explore the viewpoints of professional experts from health, educational, and social spheres about children’s health assets, access to them, and proposals for improvement. All these factors were contextualised in a specific community, following the recommendations for health promotion research [3,7].

Based on these objectives, the results obtained after the analysis of the data are organised into two blocks: (1) the perspective of experts regarding the identification of and access to assets for children’s health and well-being, and (2) proposals from professionals to promote children’s health and well-being.

### 3.1. Perspective of Experts Regarding the Identification of and Access to Assets for Children’s Health and Well-Being

#### 3.1.1. Identifying Assets for Children’s Health and Well-Being

The participants indicated that the neighbourhood is a good environment for the healthy and happy growth of children, which coincides with the opinions of the families in the same neighbourhood [30]. However, they pointed out that, among the different areas, some enhance health and well-being more than others and that not all children in the neighbourhood have favourable environments, noting the great differences that exist:

*“In this classroom there are girls who are going on vacation to Thailand and others who have barely left the neighbourhood.”*

(MP1. Primary Teacher. Female)

The experts expressed various reasons for considering whether or not Torrero-La Paz is a good neighbourhood for children (See Table S1 in Supplementary Materials).

The “determinants of healthy and happy growth” were considered to be important to very important/fundamental with the exception of “having talent in something” and “discipline”. The most valued were “physical security (feeling safe)”, “access to education” and “access to healthcare”, followed by “smooth and peaceful communication in the family”, “having people who love them very much”, “decent housing”, “doing activities that are important to them”, “access to culture”, and “economic security (having basic needs covered)”, in that order.

The participating experts added other aspects that they considered determinants of healthy and happy childhood growth, which were compiled to reach a consensus. The most important/fundamental for healthy and happy childhood growth was “economic security (having basic needs met)” followed by “physical security (feeling safe)” and “having people who love them very much”. However, participants noted the difficulty of choosing only one determinant, alluding to the complexity of the context and the interrelationship and interaction between determinants:

*“It is very difficult to select just one, taking into consideration the cultural and socioeconomic differences of the families in the neighbourhood.”*

(TL1. Leisure and free time monitor. Female)

*“I consider that there is not only one very important aspect, but there are several that converge to positively influence health and well-being.”*

(TL2. Leisure and free time monitor. Female)

*“It is difficult to consider only one aspect, so I have forced myself to prioritise the economic aspect over other areas that are also fundamental.”*

(Psychologist. Female)

**Figure 2:**
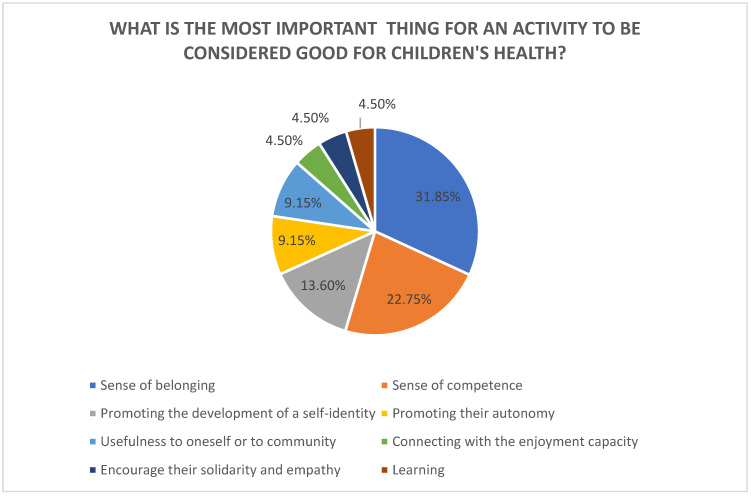
The most important aspects for an activity to be beneficial to health.

Regarding occupations, all participants related them to childhood health and well-being, which is in line with one of the bases of occupational therapy and reinforces the consideration of occupations as assets for health [30,43]. For an activity to be considered beneficial for health and development, all aspects of the form were considered important, in the following order: first, that it encourages their solidarity and their autonomy and that it makes their family feel proud and learning; second, that it allows them to develop their creativity and a sense of belonging to a group; and third, performing activities with friends and a sense of competence (feeling that he/she is doing well), with the least important item being “that the activity allows him/her to leave the family environment”. The participants also proposed other aspects that were added to the second round for prioritisation, the results of which are shown in Figure 2.

Thus, for participation in activities to be beneficial for children, great importance was given to belonging, social participation with friends, and the approval and support of their families, which reveals dimensions that go far beyond performing the duties of an occupation, and this is in line with approaches based on an understanding that human beings do with and for others [44]. The experts highlighted other aspects for an activity to be beneficial that connect with the internal assets identified by Whiting et al. [31] such as “pride”, “having fun”, “self-identity”, being physically active, “developing child”, and “I’m growing up”.

In addition to access to activities that foster children’s development, support networks and mutual help were also identified as assets, which can be related to the studies reporting that children and adolescents value components that generate social capital, linked to the community’s capacity to face adversity together and mobilise its own resources [6,33]. In the second place, green areas and resources and services related to the participants’ work were noted, something that also coincides with the results of previous research with professionals [36].

On the other hand, the worst evaluated aspects were streets and housing, and the most deficient aspects were the possibility of a hopeful future and emotional support within the family. The family is considered a basic and fundamental unit, which may be related to the findings of previous studies carried out with children themselves, identifying the family and belonging to it (“being a family”) as a stabilising asset [31]. Hernán et al. [45] incorporated into the asset model the dimensions of parental interaction, developed in attachment theory, to build a secure foundation for childhood development, which include availability, responsiveness, acceptance, cooperation, and family belonging. The professionals pointed to the importance of not only being loved but also knowing that they have love and emotional support, which is in line with the assets of “having” a personal feeling of certainty (internal asset) about the availability of resources that improve the necessary conditions to be healthy and feel good, as identified in previous studies [6].

Table 2 presents the main findings on the identification of HAs for children in Torrero-La Paz.

#### 3.1.2. Access to Health Assets

Participants more readily recognised the barriers to achieving children’s health in the neighbourhood than the assets that enhance it, which is in line with the findings of previous studies involving professionals [36,37].

The contributions of the participants in the expert panel highlight the difficulty of separating the impact of different factors that influence access to health assets, in line with the intersectional approach, which questions the hierarchisation and compartmentalisation of social markers that act as axes of inequality and oppression [22].

Even so, there was an important consensus in considering poverty or scarce economic resources, together with low social status, as one of the main barriers to accessing health assets and well-being. This barrier intersects with others, also identified by the participants, such as cultural or linguistic barriers, the lack of information and education in families, and the scarcity of healthcare services and resources in the neighbourhood, which coincides with the findings of previous studies with professionals [36,37].

The results consistently reflected the great inequities that exist between children living in the same context, which are manifested both in the physical and housing environments, with significant differences between areas that may be contiguous, and in the differences in their family’s emotional support environment and the possibility of a hopeful future. Previous studies on children’s health assets that capture these inequities in the same neighbourhood were not found.

What is consistent with previous studies is that, among the barriers to children’s access to assets and health, the participants highlighted the lack of access to health services, the lack of information and education for poorer families [36,37], poverty [36,37], background, and cultural and linguistic barriers [37]. They also pointed to streets and housing conditions as obstacles, something that is reflected, among other factors, in previous publications on the influence of neighbourhoods on health [46,47,48].

*“Mothers give juice to their children because they think it is the best and spend money they don’t have.”*

(MC1. Intercultural mediator. Female)

*“Many children don’t use the resources available because families don’t know about them.”*

(TS1. Social worker. Female)

*“We have a problem in reaching families that has to do with cultural and often linguistic barriers.”*

(MF1. General practitioner. Female)

*“Excess of population linked to the same health centre, few paediatricians, scarce civic centre, population with social difficulties that we don’t work with.”*

(TL3. Leisure and free time monitor. Female)

*“There are areas where the streets and houses do not meet the conditions for the proper development and health of children.”*

(ES2. Social educator. Male)

With respect to family-related barriers, the main barrier identified for access to child health and well-being was violence within the family for reasons exemplified by statements such as the following:

*“Violence generates situations that are incompatible with well-being, in many cases permanent, and builds mental and behavioural patterns that reproduce the violence itself.”*

(MP3. Primary school teacher. Male)

*“When their own integrity is at risk It is not possible to work with the rest of the spheres.”*

(MP5. Primary school teacher. Male)

*“Exposure to violence in the family environment seems to me to be a very serious limiting factor for the healthy (biopsychosocial) development of people. At the same level of seriousness as poverty or lack of economic resources.”*

(MF1. General practitioner. Female)

This is in line with research on the adverse effects of child abuse and neglect that can last a lifetime, such as developmental delay and impairment; physical injury; impaired reading and perceptual reasoning; depression; anxiety; post-traumatic stress disorder; low self-esteem; drug and alcohol use; aggression; and school performance deficits [49,50,51,52].

In line with the identification of the family and belonging as a stabilising asset [31] and the importance of family interaction as a secure basis for healthy childhood development [17], the experts found barriers and difficulties within families for access to health and well-being and associated this with the other factors already mentioned:

*“The fact of having low economic resources means that families focus their resources on other things rather than on the health of their children in order to survive.”*

(TL1. Leisure and free time monitor. Female)

*“The need to take care of other dependent family members; dysfunctional educational styles, poor couple relationships; lack of affectivity in the family.”*

(P1. Paediatrician. Male)

*“Family problems (disintegration, abandonment, addictions…).”*

(MP3. Primary school teacher. Male)

*“Lack of availability of caregivers.”*

(AI1. Infant classroom assistant. Female)

All the expert panel’s comments on the barriers inhibiting access to health and well-being for children in this neighbourhood, organised into categories, can be found in Table S2 of Supplementary Materials.

Difficulties were also identified in children’s access to healthcare associated with the origin of the children themselves or their families and with ethnicity. In migrant children, and even in those born in Spain but with migrant parents, more difficulties were identified in children from Africa. These barriers can be associated with racism, which, in line with the study by Rodríguez-García et al. [26], persists as a strong axis of stigmatisation and inequality in the collective imaginary, influencing access to assets for racialised children in the neighbourhood. It is necessary to explore this issue further in future research.

Roma children were noted to have the same level of difficulty in accessing healthcare. However, in this case, this difficulty is attributed to “culture”, “idiosyncrasy”, and “self-marginalisation”. This shows the persistence in the discourse of the blocking model, which overemphasises the cultural factor and attributes any imbalance or difficulty to culture and supposed ancestral inheritance and customs [25]. However, there is no consensus on this point, as some participants also reflect the historical discrimination to which the Roma people have been exposed [25]. The “openness model” regarding cultural competence, which implies the awareness of the importance of interculturality, constant questioning, proactivity, bidirectionality, and partnerships with the Roma community [25], is reflected above all in the proposals for action, which will be discussed in the subsequent section. Table 3 lists the reasons attributed by the experts participating in the panel for the difficulty of access to healthcare for migrant and Roma children.

The main barriers, identified by professionals, regarding access to health and assets for children in the neighbourhood are summarised in Table 4.

The impact of gender on access to health assets deserves to be explained in a separate section since there was no consensus among participants. Notably, 40.9% of experts considered that the fact of being a girl, on its own, without other inequality factors or oppression axes, may constitute a barrier to access to health and well-being, for reasons such as the following:

*“Girls, although not in all cases, are treated in a biased way because they are girls. We have a long way to go in the field of coeducation.”*

(TL3. Leisure and free time monitor. Female)

*“They are (or will be) affected by the patriarchal bias that characterises our society.”*

(MP3. Primary school teacher. Male)

By contrast, 59.1% felt that girls have the same opportunities to access health and well-being as boys in the absence of other socioeconomic factors that hinder access, citing reasons such as the following:

*“They have the same opportunities.”*

(P1. Paediatrician. Male)

*“There is equal treatment.”*

(EC2. Street educator. Female)

From the intersectional perspective [22], gender and the roles and mandates assigned to it are not considered barriers to accessing health and well-being assets in isolation, and they should be considered together with other factors or markers. However, the fact that the participants did not consider this factor to have any influence on inequities contradicts previous publications and studies that have identified gender as one of the axes of health inequities to be considered for incorporation into community health research and strategies [21,22]. Among the results of a previous study on children’s HAs in Torrero-La Paz is the influence of gender in the identification of and access to HAs [30]. We can explain this dissonance by contextualising it in the present time and in the sociopolitical context that the participating experts are part of. This is an era in which men’s power and social privilege are articulated in different ways, and there is a perceived gender equality. This perception has to do with the premise that “there is no more gender discrimination in the West” [53]. This is closely linked to the concept of “gender ideology”, which is widespread throughout the world and whose origins date back to the 1990s in the Catholic Church to confront the advance of feminism, and the transformation that this could bring about in the organisation of sexuality, reproduction, and the institution of family [54,55].

### 3.2. Proposals from Professionals to Promote Children’s Health and Well-Being

The experts contributed reflections and ideas for improving the neighbourhood for children in the following dimensions: (A) coexistence, (B) equal opportunities, (C) accessibility to activities and resources, (D) safety and autonomy, (E) spaces and places, and (F) other proposals. These dimensions are listed in Table S3 in Supplementary Materials. The participants were asked about the importance of improving several aspects to positively influence children’s health and well-being. Overall, 50% of the participants considered that the first improvement to be made should be “specific projects for families and projects in partnership with associations of disadvantaged groups” and “associations to promote leisure and health”, followed by (2) “more education for families” and “active schools”, (3) “More social services” and “increase social/community participation”, and (4) “more coordination and collaboration”. In addition, they also considered “understanding well-being not as having more, but as being and feeling better” and “budget for infrastructure” as important starting points. The need to improve services and infrastructure budgets is in line with previous studies on assets for health carried out with professionals [36,37].

Thus, the experts prioritised **work with families and education, including the concept of well-being and health, and collaboration with vulnerable groups and community participation** were considered key factors. All of these findings suggest a discourse in line with the model of openness that implies proactivity, outreach to the community and a willingness to change views and to understand and incorporate interculturality [25]. The participants also proposed actions such as **guaranteeing information and access to all sectors of the population**, “study and training grants”, and “free early childhood education”, among others.

The concrete proposals involving the different services and resources shared through this research were valued as real, adequate, and possible, reflecting the high involvement and motivation of the professionals working with children in the neighbourhood and **the need to give visibility and support to the actions that are already being carried out**. This last result coincides with the findings of another study in a rural community [38]. The previous study also highlighted the importance of **improving and deepening coordination between the different services,** as well as **planning and monitoring actions**. Likewise, the participants in the current study indicated the need to **take into consideration socioeconomic aspects and infrastructure and the need for support from policymakers** to improve services and made specific proposals for working with families:

*“These proposals suggest to me that there are many professionals and people involved in the neighbourhood who want to improve things and that more visibility should be given to all of this.”*

(TL3. Leisure and free time monitor. Female)

*“They seem to me to be proposals that go in the right direction, but they require in-depth, participatory and integrated planning work that does not lose sight of aspects such as urban planning or those that go beyond the neighbourhood, such as the socioeconomic situation and structure.”*

(MP3. Primary school teacher. Male)

*“For me, the most important thing would be to redouble our efforts in working with families.”*

(EC1. Street educator. Male)

*“I think all the proposals are very good, but there is a lack of financial and human resources to carry them out.”*

(MI2. Infant school teacher. Female)

In addition, the participants noted that there are demands beyond the context of the neighbourhood that have to do with guaranteeing “decent economic resources”, “housing”, and “employment”.

These results are aligned with the principles of community health and asset-based approaches [9,10] that highlight the need to promote the agency of people and communities in the management and control of their health; to enhance networks, including openness, collaboration with the community, and supporting initiatives that are already being carried out; to deepen coordination; and to provide a budget, through greater support from administrations, for improving public policies to ensure access to health and dignified living for all sectors of the population. All the above suggestions also coincide with the findings of previous studies with the participation of professionals [8,37].

### 3.3. Limitations and Strengths

The main strengths of this study are based on what it adds to children’s HA research contextualised in a neighbourhood. The study fills an existing gap in research on this topic, as it gathers the opinions of professional experts on children and families. Another strength we can highlight is that the heterogeneity of the neighbourhood allows us to verify inequities in children’s access to health assets, and it also identifies the social and cultural determinants of these inequities that intersect with each other in this community. None of these aspects could be found in previous studies on HAs in a neighbourhood (neither with children nor with adults). Another novel contribution is that this research delves into the interrelationship between HAs and occupations from the perspective of occupational therapy.

Another strength is that the expert participants are representative of all areas and roles of intervention with children and families in this community. Furthermore, the study goes beyond internal assets and delves into the interrelationship between assets, social capital, and community development, as recommended in the specialised literature.

Our findings allow us to delve deeper into the identification of children’s health assets in this neighbourhood and the barriers and facilitators for access to them. Furthermore, this study provides concrete and real proposals for improvement from professionals with extensive experience working with both children and families in this local context. For future research, it would be advisable to continue gathering not only the opinions of professionals but also of children themselves, as well as their families.

A limitation of the Delphi technique is the risk of bias in the selection of the expert group. In this study, the number of participants was small, and they all expressed interest in participating in the research process. Therefore, other experts who were not available to participate were not considered.

## 4. Conclusions

This study highlights the perspective of professionals on health assets and the proposals of experts in working with children and families in a local community. This study supports previous research on child and youth HAs in identifying assets for child health, as it highlights support and mutual aid networks; green spaces; and educational, health, and social resources and services as key HAs. The results showing the linkage of children’s health and well-being with the availability of unconditional emotional support networks, especially family, and the sense of certainty of having them, also reinforce the findings of previous research on childhood and youth HAs and the theories and models of mental and emotional health during childhood.

Meaningful occupations are also revealed as assets that promote children’s health and well-being, which supports the basic principles of occupational therapy.

Our study supports the premises of the social determinants of health framework and the intersectional perspective. There are great inequities between different boys and girls living in the same neighbourhood, not only in terms of access to HAs but also health and well-being in general. These inequities are determined by several interacting and intersecting factors such as poverty, origin, ethnicity, social status, and gender. 

The proposals to promote the health and well-being of children focus above all on valuing, supporting, and reinforcing the assets that already exist, as well as strengthening collaboration and coordination, not only between professional resources but also with the community and, above all, with the most vulnerable groups. This supports the strategies and proposals of the openness model towards intercultural competence and community health and asset-based approaches. Other proposals to improve child health in Torrero-La Paz include working with families and in schools; guaranteeing information and access to all sectors of the population through grants and free public services; giving visibility and support to actions that are already being carried out; the planning and monitoring of actions; improving infrastructures; and increasing the support from policymakers; taking into account socioeconomic factors; and guaranteeing decent economic resources, housing, and employment.

## Figures and Tables

**Table 1:** Experts participating in the panel.

Tool	Gender/n (%)	Sector/n (%)	Users/n	Experience in Years/n (%)
Interviews n = 3	Female/2 (66.7%) Male/1 (33.3%)	Formal Education/1 (33.3%) Social/1 (33.3%) Healthcare/1 (33.3%)	Children and families/2 Families/1	More than 7/3 (100%)
Delphi n = 22	Female /15 (68.2%) Male/7 (31.8%)	Formal education: 8 (36.4%) Non-formal education/5 (22.7%) Social/5 (22.7%) Healthcare/4 (18.2%)	Children/12 Families/4 Children and families/6	1.5–2 = 2 (9.1%) 2–3 = 2 (9.1%) 4–5 = 3 (13.6%) 6–7 = 4 (18.2%) More than 7 = 11 (50%)

**Table 2:** Torrero-La Paz children’s health asset identification.

Places and Resources	Main Determinants of Healthy and Happy Childhood Growth	Internal HAs Related to Occupations	Social and Emotional Support
Green areas	Economic security	Sense of belonging	Family
Accessible schools	Physical security	Sense of competence	Support networks
Social resources	Love and emotional support	Self-identity	Mutual help
Sports areas		Autonomy	
Small commerce		Ability to perform useful activities	
Leisure resources			

**Table 3:** Reasons for the difficulty of access to health and welfare for migrant and Roma children.

Migrant and Migrant-Parent Children	Roma Children
*“Difficulties with the **language**, they do **not trust** the health system.”* (EC2. Street educator. Female) *“Migrant families have **less historical background** in the neighbourhood and therefore may be unaware of or have less access to resources.”* (TL2. Leisure and free time monitor. Female) *“Difficulties with **language**.”* (PT1. Therapeutic pedagogy teacher. Female) *“**Economic**, **language** and **cultural** difficulties.”* (MF1. General practitioner. Female) *“Because sometimes their cards are linked to those of their parents and if the latter **do not have social security**, the problem sometimes arises.”* (PS1. Psychologist. Female) *“There are migrant families with greater difficulties due to **language and cultural codes**.”* (TS1. Social worker. Female) *“Sometimes because of **language** problems. Sometimes due to their **irregular situation** or **lack of knowledge** of the services they can access.”* (MI1. Infant school teacher. Female) *“**Fears** and **lack of knowledge** of the system.”* (ES1. Social educator. Male) *“They are **less rooted** in the neighbourhood and **culture**.”* (MP4. Primary school teacher. Male)	*“Because of the **idiosyncrasies** of their culture and the difficulty of leaving it behind.”* (P1. Paediatrician. Male) *“I think that the Roma in general are the ones who have the hardest time, as **their cultural perception** does not make them value access to health and child welfare in the same way as us.”* (EC1. Street educator. Male) *“Having **decent housing** facilitates health and well-being and many Roma families do not have this.”* (MI1. Intercultural mediator. Female) *“I think that in many cases they have very **chronic situations** with a **lack of adaptation**.”* (TL3. Leisure and free time monitor. Female) *“I have the impression that in the Roma community there are some **patterns or habits**, perhaps typical of a **closed community** (with nuances) and **marginalised** for generations, that do not value the health and well-being of its members.”* (MP3. Primary school teacher. Male) *“Roma families are aware of the resources and make use of them, although **they need** certain **socioeducational and socioemotional guidelines**.”* (TS1. Social worker. Female) *“They are very wary of going to services and do **not like to be controlled**.”* (EC1. Street educator. Male) *“Due to their own **self-marginalisation**.”* (MP5. Primary school teacher. Male)

**Table 4:** Barriers to children’s access to health.

Barriers
Poverty
Low social status
Barriers within the family: lack of information and education, violence, shortage of emotional support and affectivity
Shortage of healthcare services
Lack of adequate physical environments and decent housing
Being a migrant or the son or daughter of migrants (especially Afro-descendants)
Belonging to the Roma people

## Data Availability

The data presented in this study are available on request from the corresponding author.

## Supplementary Materials

The following supporting information can be downloaded at: https://www.mdpi.com/article/10.3390/healthcare12050506/s1, Script S1: In-depth interview script; Round 1 Form S1: Questionnaire for the first Delphi round; Round 2 Form S2: Questionnaire for the second Delphi round; Table S1: Reasons why Torrero is or is not a good neighbourhood for children to grow up healthy and happy. Table S2: Barriers to children’s access to health and well-being; Table S3: Improvement proposals for the promotion of children’s health in the neighbourhood.

## References

[1] World Health Organization (1986). The Ottawa Charter for Health Promotion.

[2] Antonovsky A. (1996). The Salutogenic Model as a Theory to Guide Health Promotion1. Health Promot. Int..

[3] Morgan A., Hernán M. (2013). Promoting Health and Wellbeing through the Asset Model. Rev. Esp. Sanid. Penit..

[4] Lindström B., Eriksson M. (2009). The Salutogenic Approach to the Making of HiAP/Healthy Public Policy: Illustrated by a Case Study. Glob. Health Promot..

[5] Morgan A., Ziglio E. (2007). Revitalising the Evidence Base for Public Health: An Assets Model. Promot. Educ..

[6] Pérez-Wilson P., Hernán M., Morgan A.R., Mena A. (2015). Health Assets for Adolescents: Opinions from a Neighbourhood in Spain. Health Promot. Int..

[7] WHO First International Conference on Health Promotion, Ottawa, 21 November 1986. https://www.who.int/teams/health-promotion/enhanced-wellbeing/first-global-conference.

[8] Pons-Vigués M., Berenguera A., Coma-Auli N., Pombo-Ramos H., March S., Asensio-Martínez A., Moreno-Peral P., Mora-Simón S., Martínez-Andrés M., Pujol-Ribera E. (2017). Health-Care Users, Key Community Informants and Primary Health Care Workers’ Views on Health, Health Promotion, Health Assets and Deficits: Qualitative Study in Seven Spanish Regions. Int. J. Equity Health.

[9] Peña P.A., Azagra C.B.B. (2019). Conversando: Sobrediagnóstico, sobretratamiento y recomendación de activos para la salud. Comunidad.

[10] Van Bortel T., Wickramasinghe N.D., Morgan A., Martin S. (2019). Health Assets in a Global Context: A Systematic Review of the Literature. BMJ Open.

[11] Alvarez-Dardet C., Morgan A., Cantero M.T.R., Hernán M. (2015). Improving the Evidence Base on Public Health Assets—The Way Ahead: A Proposed Research Agenda. J. Epidemiol. Community Health.

[12] Sun Q., Loveday M., Nwe S., Morris N., Boxall E. (2023). Green Social Prescribing in Practice: A Case Study of Walsall, UK. Int. J. Environ. Res. Public Health.

[13] Smyth N., Thorn L., Wood C., Hall D., Lister C. (2022). Increased Wellbeing Following Engagement in a Group Nature-Based Programme: The Green Gym Programme Delivered by the Conservation Volunteers. Healthcare.

[14] Bradley G., Scott J. (2021). Social Prescribing Nomenclature, Occupational Therapy and the Theory of Institutional Work: Creating, Maintaining and Disrupting Medical Dominance. Occup. Ther. Health Care.

[15] Commission on the Social Determinants of Health (2008). Closing the Gap within a Generation: Health Equity through Action on the Social Determinants of Health. Final Report of the Commission on Social Determinants of Health.

[16] Marmot M. (2005). Social Determinants of Health Inequalities. Lancet.

[17] Pillas D., Marmot M., Naicker K., Goldblatt P., Morrison J., Pikhart H. (2014). Social Inequalities in Early Childhood Health and Development: A European-Wide Systematic Review. Pediatr. Res..

[18] Solar O., Irwin A. (2010). A Conceptual Framework for Action on the Social Determinants of Health. Social Determinants of Health Discussion Paper 2 (Policy and Practice).

[19] Braveman P., Egerter S., Williams D.R. (2011). The Social Determinants of Health: Coming of Age. Annu. Rev. Public Health.

[20] Evans L., Engelman M., Mikulas A., Malecki K. (2021). How Are Social Determinants of Health Integrated into Epigenetic Research? A Systematic Review. Soc. Sci. Med. 1982.

[21] Artazcoz L., Chilet E., Escartín P., Fernández A. (2018). Incorporación de La Perspectiva de Género En La Salud Comunitaria. Informe SESPAS 2018. Gac. Sanit..

[22] Couto M.T., de Oliveira E., Separavich M.A.A., Luiz O.d.C. (2019). La Perspectiva Feminista de La Interseccionalidad En El Campo de La Salud Pública: Revisión Narrativa de Las Producciones Teórico-Metodológicas. Salud Colect..

[23] Font-Ribera L., García-Continente X., Davó-Blanes M.C., Ariza C., Díez E., García Calvente M.d.M., Maroto G., Suárez M., Rajmil L. (2014). El Estudio de Las Desigualdades Sociales En La Salud Infantil y Adolescente En España. Gac. Sanit..

[24] González-Rábago Y., Martín U. (2019). Salud y Determinantes Sociales de La Salud En Hijos e Hijas de Personas Inmigrantes Internacionales: ¿desigualdades Sociales En Salud Desde La Infancia?. Gac. Sanit..

[25] Arza-Porras J., Félix Rodríguez-Camacho M. (2019). Competencia Intercultural Profesional En La Atención Socio-Sanitaria a La Población Gitana En España. Altern. Cuad. Trab. Soc..

[26] Rodríguez-García D., Jordana T.H., Reche C.R. (2021). “Tú, Como Eres Negra, Harás de Lobo”. El Debate Pendiente Sobre La Cuestión de La Raza En España. Perif. Rev. Investig. Form. Antropol..

[27] Baum F. (2017). Health Divides. Where You Live Can Kill You. Int. J. Epidemiol..

[28] Starfield B., Robertson J., Riley A.W. (2002). Social Class Gradients and Health in Childhood. Ambul. Pediatr. Off. J. Ambul. Pediatr. Assoc..

[29] Hernán M., Morgan A., Mena A. (2018). Formación En Salutogénesis y Activos Para La Salud|Escuela Andaluza de Salud Pública.

[30] Vidal-Sánchez M.I., Laborda-Soriano A.A., Cambra-Aliaga A., Sanz-Valer P., Gasch Gallén Á. (2021). Childhood Health Assets in a Spanish Neighborhood: Children and Families’ Perception. OTJR Occup. Particip. Health.

[31] Whiting L.S., Kendall S., Wills W. (2013). Rethinking Children’s Public Health: The Development of an Assets Model. Crit. Public Health.

[32] Calmeiro L., Camacho I., Matos M.G.d. (2018). Life Satisfaction in Adolescents: The Role of Individual and Social Health Assets. Span. J. Psychol..

[33] Eriksson M., Dahlblom K. (2020). Children’s Perspectives on Health-Promoting Living Environments: The Significance of Social Capital. Soc. Sci. Med..

[34] Hammell K.W. (2019). Building Globally Relevant Occupational Therapy from the Strength of Our Diversity. World Fed. Occup. Ther. Bull..

[35] Wilcock A.A., Hocking C. (2015). An Occupational Perspective of Health.

[36] Den Broeder L., Uiters E., Hofland A., Wagemakers A., Schuit A.J. (2017). Local Professionals’ Perceptions of Health Assets in a Low-SES Dutch Neighbourhood: A Qualitative Study. BMC Public Health.

[37] Thein K., Thuya Zaw K., Teng R.-E., Liang C., Julliard K. (2009). Health Needs in Brooklyn’s Chinatown: A Pilot Assessment Using Rapid Participatory Appraisal. J. Health Care Poor Underserved.

[38] Alberdi-Erice M.J., Rayón-Valpuesta E., Martinez H. (2022). Promoting Health in a Rural Community in the Basque Country by Leveraging Health Assets Identified through a Community Health Diagnosis. Int. J. Environ. Res. Public Health.

[39] García Valdés M., Suárez Marín M. (2013). El Método Delphi Para La Consulta a Expertos En La Investigación Científica. Rev. Cuba. Salud Pública.

[40] Barrett D., Heale R. (2020). What Are Delphi Studies?. Evid. Based Nurs..

[41] Winkler D., Unsworth C., Sloan S. (2005). Time Use Following a Severe Traumatic Brain Injury. J. Occup. Sci..

[42] Nasa P., Jain R., Juneja D. (2021). Delphi Methodology in Healthcare Research: How to Decide Its Appropriateness. World J. Methodol..

[43] Alsina-Santana R., Zango-Martín I. (2022). El Abordaje de Terapia Ocupacional Considerando Los Activos Para La Salud En Población Joven: Un Análisis de La Literatura. Cad. Bras. Ter. Ocup..

[44] Hocking C. (2020). Occupation in Context: A Reflection on Environmental Influences on Human Doing. J. Occup. Sci..

[45] Cofiño R., Aviñó D., Benedé C.B., Botello B., Cubillo J., Morgan A., Paredes-Carbonell J.J., Hernán M. (2016). Promoción de La Salud Basada En Activos: ¿cómo Trabajar Con Esta Perspectiva En Intervenciones Locales?. Gac. Sanit..

[46] Bambra C. (2016). Health Divides: Where You Live Can Kill You.

[47] Fariña Tojo J., Higueras García E., Román López E. Ciudad, Urbanismo y Salud. Documento Técnico de Criterios Generales Sobre Parámetros de Diseño Urbano Para Alcanzar los Objetivos de una Ciudad Saludable Con Especial Énfasis en el Envejecimiento Activo. https://oa.upm.es/65377/.

[48] Odgers C.L., Moffitt T.E., Tach L.M., Sampson R.J., Taylor A., Matthews C.L., Caspi A. (2009). The Protective Effects of Neighborhood Collective Efficacy on British Children Growing Up in Deprivation: A Developmental Analysis. Dev. Psychol..

[49] Bellis M.A., Hughes K., Ford K., Ramos Rodriguez G., Sethi D., Passmore J. (2019). Life Course Health Consequences and Associated Annual Costs of Adverse Childhood Experiences across Europe and North America: A Systematic Review and Meta-Analysis. Lancet Public Health.

[50] Hughes K., Bellis M.A., Hardcastle K.A., Sethi D., Butchart A., Mikton C., Jones L., Dunne M.P. (2017). The Effect of Multiple Adverse Childhood Experiences on Health: A Systematic Review and Meta-Analysis. Lancet Public Health.

[51] Maguire-Jack K., Lanier P., Johnson-Motoyama M., Welch H., Dineen M. (2015). Geographic Variation in Racial Disparities in Child Maltreatment: The Influence of County Poverty and Population Density. Child Abuse Negl..

[52] Taillieu T.L., Brownridge D.A., Sareen J., Afifi T.O. (2016). Childhood Emotional Maltreatment and Mental Disorders: Results from a Nationally Representative Adult Sample from the United States. Child Abuse Negl..

[53] Alonso A., Espinosa-Fajardo J. (2021). Blitzkrieg Against Democracy: Gender Equality and the Rise of the Populist Radical Right in Spain. Soc. Polit. Int. Stud. Gend. State Soc..

[54] Case M.A. (2019). Trans Formations in the Vatican’s War on “Gender Ideology. ” Signs J. Women Cult. Soc..

[55] Sánchez L.A. (2022). El marco de la «ideología de género» en el discurso de Vox. Más Poder Local.

