# Supplementary material for: Professional Perspectives on Children’s Health Assets: A Delphi Study
Source: Healthcare (Basel). 2024 Feb 20;12(5):506. doi: 10.3390/healthcare12050506 (PMC10930817; doi:10.3390/healthcare12050506)
Supplement: Supplementary file 1 [file healthcare-12-00506-s001.zip › healthcare-2785873-supplementary.pdf]

Script 1 S1: In-depth interview Script

1. Personal data: age, gender, occupation
2. What is your connection with this neighbourhood and since when?
3. Why were you interested in conducting this interview?
4. What is health for you? *If he/she does not include it: ask about emotional/mental and social health.
5. What do you think is the most important thing for children's health?
6. What do you think the mental health of children in the neighbourhood is like?
7. What is there in this neighbourhood that is good for children's health? What is missing?
8. Ask about the importance for children's health of:
   The family
   Neighbourhood relations
   Dignified and healthy housing
   Streets
   Parks
   Social movements
   The Health Centre
   Health professionals
   Social services
   The school
   Teachers
   The Civic Centre
   The Municipal Sports Centre
   The associative movement (parents', youth, neighbourhood associations, etc.).
   Sports groups, mutual aid groups, religious groups,.. (Name them)
   The Library
   The shops. Which ones? ___________________________________
   More: ___________________
9. What do you think needs to be done to improve children's mental health in the neighbourhood? Where should we start? Who should we involve? What networks should we build or strengthen?
10. Can you name key actors or experts in child's health and well-being in this neighbourhood?
11. Add anything you want

Round 1 Form S1: Questionnaire for the first Delphi round

1/12/23, 19:16 CUESTIONARIO 1. DELPHI ACTIVOS INFANCIA TORRERO-LA PAZ

CUESTIONARIO 1. DELPHI ACTIVOS INFANCIA TORRERO-LA PAZ

Este estudio forma parte de la Investigación "Desigualdades sociales y de género en salud en la infancia. El papel del capital social y los activos en salud", aprobada por el Comité de Ética en la Investigación de Aragón.

El principal objetivo de esta fase, en la que usted participa, es identificar los activos para la salud (fortalezas y recursos que mejoran y mantienen la salud y que ayudan a acabar con las desigualdades en salud) de la infancia del barrio y las estrategias que podrían fomentarlos.

En un primer momento el equipo investigador debe poder identificar a los y las informantes clave que participan en el Delphi para poder registrar quiénes responden y realizar las siguientes vueltas; también se le solicitará el correo electrónico para que, si lo desea, pueda recibir una copia de sus respuestas. Toda la información recogida se tratará conforme a lo establecido en la legislación vigente en materia de protección de datos de carácter personal. En la base de datos del estudio no se incluirán datos personales: ni su nombre, ni ningún dato que le pueda identificar. Se le identificará por un código. Sólo el equipo investigador tendrá acceso a sus datos y nadie ajeno a la investigación podrá consultarlos.
Las conclusiones del estudio se presentarán en congresos y publicaciones científicas, pero se harán siempre con datos agrupados y nunca se divulgará nada que le pueda identificar.

Es importante que NO comparta sus respuestas e impresiones con otras personas que participan en el Delphi hasta que hayamos finalizado la recogida de respuestas de todas las fases
Del mismo modo, es importante que, si se compromete a participar, lo haga hasta el final de este estudio

PARA CUALQUIER DUDA. INCIDENCIA O CONSULTA PUEDE CONTACTAR CON LA INVESTIGADORA PRINCIPAL:
Isabel Vidal Sánchez.
Mail: vidal@unizar.es
Teléfono: 666914929

* Indica que la pregunta es obligatoria

1. Correo *

____

https://docs.google.com/forms/d/1KBzGHahzkUI3JluP1Y02N6Y1nERYm3VqhHPv4pJB7UY/edit#question=162577701&field=1701703616 1/27

1/12/23, 19:17 CUESTIONARIO 1. DELPHI ACTIVOS INFANCIA TORRERO-LA PAZ

2. He recibido suficiente información sobre el estudio y comprendo que mi participación es voluntaria: *

Marca solo un óvalo.

- ◯ Sí

DATOS PARTICIPANTE

3. *Este dato sólo se recoge para facilitar las siguientes vueltas del Delphi y aseguramos de que sólo participan informantes clave. Al analizar los resultados se anonimizarán* NOMBRE:

____

4. Es usted:

Marca solo un óvalo.

- ◯ Mujer
- ◯ Hombre
- ◯ Otro: ____________

5. ÁMBITO EN EL QUE INTERVIENE (marcar sólo uno, el que más represente su labor)

Marca solo un óvalo.

- ◯ SOCIAL
- ◯ SANITARIO
- ◯ EDUCACIÓN FORMAL
- ◯ EDUCACIÓN NO FORMAL

6. PROFESIÓN

____

https://docs.google.com/forms/d/1KBzGHahzkUI3JluP1Y02N6Y1nERYm3VqhHPv4pJB7UY/edit#question=162577701&field=1701703616 2/27

1/12/23, 19:17 CUESTIONARIO 1. DELPHI ACTIVOS INFANCIA TORRERO-LA PAZ

7. POBLACIÓN CON LA QUE DESEMPEÑA O HA DESEMPEÑADO SU LABOR EN LOS ÚLTIMOS 5 AÑOS (puede marcar más de una)

Selecciona todos los que correspondan.

- ☐ INFANCIA DE 3 A 5
- ☐ INFANCIA DE 6 A 9
- ☐ INFANCIA DE 10 A 12
- ☐ FAMILIAS
- ☐ PERSONAS ADULTAS QUE TIENEN INFANCIA A SU CARGO

8. AÑOS DE EXPERIENCIA EN TORRERO-LA PAZ

Marca solo un óvalo.

- ◯ 2-3 AÑOS
- ◯ 4-5 AÑOS
- ◯ 6-7 AÑOS
- ◯ MÁS DE 7 AÑOS
- ◯ Otro: ____________

9. RESIDE USTED EN TORRERO-LA PAZ *

Marca solo un óvalo.

- ◯ Sí
- ◯ No

10. Si ha contestado sí ¿Cuántos años lleva viviendo en el barrio?

____

CRECIMIENTO SANO Y FELIZ

https://docs.google.com/forms/d/1KBzGHahzkUI3JluP1Y02N6Y1nERYm3VqhHPv4pJB7UY/edit#question=162577701&field=1701703616 3/27

1/12/23, 19:17 CUESTIONARIO 1. DELPHI ACTIVOS INFANCIA TORRERO-LA PAZ

11. ¿Cree que Torrero-La Paz es un buen barrio para que la infancia crezca sana y feliz?

Marca solo un óvalo.

- ◯ Sí
- ◯ No
- ◯ Otro: ____________

12. Indique por qué

____

DETERMINANTES DE CRECIMIENTO SANO Y FELIZ

Puntúe del 0 al 4 la importancia de los siguientes aspectos para que niñas y niños crezcan sanos y felices, Siendo 0 NADA IMPORTANTE y 4 IMPORTANTÍSIMO/FUNDAMENTAL

13. 1. Seguridad económica (tener cubiertas las necesidades básicas) *

Marca solo un óvalo.

	0	1	2	3	4	
NAD	◯	◯	◯	◯	◯	IMPORTANTÍSIMO/ FUNDAMENTAL

https://docs.google.com/forms/d/1KBzGHahzkUI3JluP1Y02N6Y1nERYm3VqhHPv4pJB7UY/edit#question=162577701&field=1701703616 4/27

14. 2. Seguridad física (sentirse a salvo) *

Marca solo un óvalo.

0 1 2 3 4

NAD ◯ ◯ ◯ ◯ ◯ IMPORTANTÍSIMO/ FUNDAMENTAL

15. 3. Vivienda digna *

Marca solo un óvalo.

0 1 2 3 4

NAD ◯ ◯ ◯ ◯ ◯ IMPORTANTÍSIMO/FUNDAMENTAL

16. 4. Estar con su familia *

Marca solo un óvalo.

0 1 2 3 4

NAD ◯ ◯ ◯ ◯ ◯ IMPORTANTÍSIMO/FUNDAMENTAL

17. 5. Comunicación en la familia fluida y pacífica *

Marca solo un óvalo.

0 1 2 3 4

NAD ◯ ◯ ◯ ◯ ◯ IMPORTANTÍSIMO/FUNDAMENTAL

18. 6. Tener personas que le quieran mucho *

Marca solo un óvalo.

0 1 2 3 4

NAD ◯ ◯ ◯ ◯ ◯ IMPORTANTÍSIMO/FUNDAMENTAL

19. 7. Acceso a la educación *

Marca solo un óvalo.

0 1 2 3 4

NAD ◯ ◯ ◯ ◯ ◯ IMPORTANTÍSIMO/FUNDAMENTAL

20. 8. Hacer actividades que sean importantes para ellas/os *

Marca solo un óvalo.

0 1 2 3 4

NAD ◯ ◯ ◯ ◯ ◯ IMPORTANTÍSIMO/FUNDAMENTAL

21. 9. Acceso a la sanidad *

Marca solo un óvalo.

0 1 2 3 4

NAD ◯ ◯ ◯ ◯ ◯ IMPORTANTÍSIMO/FUNDAMENTAL

22. 10. Tener talento en algo (música, deporte, arte, tecnología,….) *

Marca solo un óvalo.

0 1 2 3 4

NAD ◯ ◯ ◯ ◯ ◯ IMPORTANTÍSIMO/FUNDAMENTAL

23. 11. Que su familia le anime en lo que hace bien *

Marca solo un óvalo.

0 1 2 3 4

NAD ◯ ◯ ◯ ◯ ◯ IMPORTANTÍSIMO/FUNDAMENTAL

24. 12. Disciplina *

Marca solo un óvalo.

0 1 2 3 4

NAD ◯ ◯ ◯ ◯ ◯ IMPORTANTÍSIMO/FUNDAMENTAL

25. 13. Futuro esperanzador *

Marca solo un óvalo.

0 1 2 3 4

NAD ◯ ◯ ◯ ◯ ◯ IMPORTANTÍSIMO/FUNDAMENTAL

26. 14. Muchas zonas verdes en el barrio en que crecen *

Marca solo un óvalo.

0 1 2 3 4

NAD ◯ ◯ ◯ ◯ ◯ IMPORTANTÍSIMO/FUNDAMENTAL

27. 15. Zonas de juego en el barrio en que crecen *

Marca solo un óvalo.

0 1 2 3 4

NAD ◯ ◯ ◯ ◯ ◯ IMPORTANTÍSIMO/FUNDAMENTAL

28. 16. Acceso a la cultura *

Marca solo un óvalo.

0 1 2 3 4

NAD ◯ ◯ ◯ ◯ ◯ IMPORTANTÍSIMO/FUNDAMENTAL

29. Añada los aspectos que considere importantes para el desarrollo sano y feliz de la infancia del barrio de Torrero-La Paz y que cree que no están reflejados en las cuestiones anteriores

30. En su opinión, ¿qué DIFICULTA el acceso a la salud y el bienestar de la infancia de Torrero-La Paz? Indique 4 factores y ordénelos de mayor a menor influencia: *

EL BARRIO PARA LA INFANCIA

Puntúe cómo cree que es el barrio con respecto a la infancia donde 0 es muy deficitario y 4 es excelente en cuanto a:

31. 1. Convivencia intercultural *

Marca solo un óvalo.

0 1 2 3 4

MU\ ◯ ◯ ◯ ◯ ◯ EXCELENTE

32. 2. Redes de apoyo y ayuda mutua *

Marca solo un óvalo.

0 1 2 3 4

MU\ ◯ ◯ ◯ ◯ ◯ EXCELENTE

33. 3. Servicios y recursos sociales *

Marca solo un óvalo.

0 1 2 3 4

MU\ ◯ ◯ ◯ ◯ ◯ EXCELENTE

34. 4. Servicios y recursos sanitarios *

Marca solo un óvalo.

0 1 2 3 4

MU\ ◯ ◯ ◯ ◯ ◯ EXCELENTE

35. 5. Servicios y recursos educativos *

Marca solo un óvalo.

0 1 2 3 4

MU\ ◯ ◯ ◯ ◯ ◯ EXCELENTE

36. 6. Apoyo emocional dentro de las familias *

Marca solo un óvalo.

0 1 2 3 4

MU\ ◯ ◯ ◯ ◯ ◯ EXCELENTE

37. 7. Acceso a actividades que fomenten su desarrollo *

Marca solo un óvalo.

0 1 2 3 4

MU\ ◯ ◯ ◯ ◯ ◯ EXCELENTE

38. 8. Calles *

Marca solo un óvalo.

0 1 2 3 4

MU\ ◯ ◯ ◯ ◯ ◯ EXCELENTE

39. 9. Vivienda *

Marca solo un óvalo.

0 1 2 3 4

MU\ ◯ ◯ ◯ ◯ ◯ EXCELENTE

40. 10. Zonas verdes *

Marca solo un óvalo.

0 1 2 3 4

MU\ ◯ ◯ ◯ ◯ ◯ EXCELENTE

41. 11. Acceso a la cultura *

Marca solo un óvalo.

0 1 2 3 4

MU\ ◯ ◯ ◯ ◯ ◯ EXCELENTE

42. 12. Futuro esperanzador *

Marca solo un óvalo.

0 1 2 3 4

MU\ ◯ ◯ ◯ ◯ ◯ EXCELENTE

DIFERENCIAS EN EL ACCESO

43. ¿Cree que hay diferencias significativas en el acceso a la salud y el bienestar de la infancia dependiendo de la edad? *

Marca solo un óvalo.

◯ Sí
◯ No

44. Si ha contestado SÍ ¿Quiénes tienen mayores dificultades?

45. ¿Cree que hay diferencias significativas en el acceso a la salud y el bienestar de la infancia dependiendo del género? *

Marca solo un óvalo.

◯ Sí
◯ No

46. Si ha contestado SÍ ¿Quiénes tienen mayores dificultades?

47. ¿Cree que hay diferencias significativas en el acceso a la salud y el bienestar de la infancia dependiendo de la etnia? *

Marca solo un óvalo.

- Sí
- No

48. Si ha contestado SÍ ¿Quiénes tienen mayores dificultades?

49. ¿Cree que hay diferencias significativas en el acceso a la salud y el bienestar de la infancia dependiendo de la procedencia/origen? *

Marca solo un óvalo.

- Sí
- No

50. Si ha contestado SÍ ¿Quiénes tienen mayores dificultades?

51. ¿Cree que hay diferencias significativas en el acceso a la salud y el bienestar de la infancia dependiendo del estatus socio-económico? *

Marca solo un óvalo.

- Sí
- No

52. Si ha contestado SÍ ¿Quiénes tienen mayores dificultades?

53. Si considera que hay algún otro factor que condiciona el acceso a la salud y bienestar de las niñas y niños de Torrero que no ha sido reflejado por favor indíquelo/s

OCUPACIONES EN LA INFANCIA

Señale la importancia de los siguientes aspectos para que una actividad sea beneficiosa para el desarrollo y la salud emocional y mental de las niñas y niños:

54. Hacer la actividad con amig@s *

Marca solo un óvalo.

0 1 2 3 4

NAD ◯ ◯ ◯ ◯ ◯ IMPORTANTÍSIMO

55. Sentido de competencia (sentir que lo hace bien) *

Marca solo un óvalo.

0 1 2 3 4

NAD ◯ ◯ ◯ ◯ ◯ IMPORTANTÍSIMO

56. Sentimiento de pertenencia a un grupo *

Marca solo un óvalo.

0 1 2 3 4

NAD ◯ ◯ ◯ ◯ ◯ IMPORTANTÍSIMO

57. Que su familia se sienta orgullosa *

Marca solo un óvalo.

0 1 2 3 4

NAD ◯ ◯ ◯ ◯ ◯ IMPORTANTÍSIMO

58. Aprendizaje *

Marca solo un óvalo.

0 1 2 3 4

NAD ◯ ◯ ◯ ◯ ◯ IMPORTANTÍSIMO

59. Que esa actividad le permita salir del entorno familiar *

Marca solo un óvalo.

0 1 2 3 4

NAD ◯ ◯ ◯ ◯ ◯ IMPORTANTÍSIMO

60. Que fomente su autonomía *

Marca solo un óvalo.

0 1 2 3 4

NAD ◯ ◯ ◯ ◯ ◯ IMPORTANTÍSIMO

61. Que fomente su solidaridad *

Marca solo un óvalo.

0 1 2 3 4

NAD ◯ ◯ ◯ ◯ ◯ IMPORTANTÍSIMO

62. Que le permita desarrollar su creatividad *

Marca solo un óvalo.

0 1 2 3 4

NAD ◯ ◯ ◯ ◯ ◯ IMPORTANTÍSIMO

63. Añada otros aspectos que considera importantes para que las actividades/cosas que hacen los niños y niñas del barrio sean buenas para su desarrollo y bienestar emocional.

¿QUIÉN LO TIENE MÁS DIFÍCIL?

Ordene del 1 al 11 la influencia de los siguientes determinantes para DIFICULTAR el acceso a la salud, el bienestar y los activos para la salud de los niños y niñas de Torrero-La Paz. Donde 1º es QUIÉN LO TIENE MÁS DIFÍCIL. *Por favor, coloque SÓLO UN ítem en cada posición*

64. Niñas

Marca solo un óvalo.

- 1º
- 2º
- 3º
- 4º
- 5º
- 6º
- 7º
- 8º
- 9º
- 10º
- 11º

65. Infancia del Pueblo Gitano

Marca solo un óvalo.

- 1º
- 2º
- 3º
- 4º
- 5º
- 6º
- 7º
- 8º
- 9º
- 10º
- 11º

66. Infancia cuya familia procede del Norte de África

Marca solo un óvalo.

- 1º
- 2º
- 3º
- 4º
- 5º
- 6º
- 7º
- 8º
- 9º
- 10º
- 11º

67. Infancia cuya familia procede del África Subsahariana

Marca solo un óvalo.

- 1º
- 2º
- 3º
- 4º
- 5º
- 6º
- 7º
- 8º
- 9º
- 10º
- 11º

68. Infancia cuya familia procede de Latinoamérica

Marca solo un óvalo.

- 1º
- 2º
- 3º
- 4º
- 5º
- 6º
- 7º
- 8º
- 9º
- 10º
- 11º

69. Infancia cuya familia procede de Asia

Marca solo un óvalo.

- 1º
- 2º
- 3º
- 4º
- 5º
- 6º
- 7º
- 8º
- 9º
- 10º
- 11º

70. Infancia cuya familia procede de Rumanía

Marca solo un óvalo.

- 1º
- 2º
- 3º
- 4º
- 5º
- 6º
- 7º
- 8º
- 9º
- 10º
- 11º

71. Niñas y niños con escasos recursos económicos

Marca solo un óvalo.

- 1º
- 2º
- 3º
- 4º
- 5º
- 6º
- 7º
- 8º
- 9º
- 10º
- 11º

72. Niñas y niños con bajo estatus social

Marca solo un óvalo.

- 1º
- 2º
- 3º
- 4º
- 5º
- 6º
- 7º
- 8º
- 9º
- 10º
- 11º

73. Infancia expuesta a violencia dentro de su ámbito familiar

Marca solo un óvalo.

- 1º
- 2º
- 3º
- 4º
- 5º
- 6º
- 7º
- 8º
- 9º
- 10º
- 11º

74. Infancia expuesta a violencia fuera de su ámbito familiar

Marca solo un óvalo.

- 1º
- 2º
- 3º
- 4º
- 5º
- 6º
- 7º
- 8º
- 9º
- 10º
- 11º

75. Por favor, explique por qué ha situado ese el 1º y añada lo que quiera respecto a este punto

PROPUESTAS DE ACCIÓN

¿Qué se podría hacer en este barrio para mejorarlo PARA LA INFANCIA? Aporte una idea que se le ocurra para cada una de estas cosas:

76. Mejorar la convivencia:

77. Igualar las oportunidades en la vida:

78. Mejorar el acceso a las cosas y actividades que les hacen felices y les hacen estar bien:

79. Fomentar la autonomía y la seguridad de niños/as en las calles:

80. Fomentar lugares agradables a los que ir y en los que estar

81. Otras sugerencias e ideas

¿Qué sería importante para mejorar el barrio para la infancia?

82. Presupuesto para infraestructuras *

Marca solo un óvalo.

	0	1	2	3	4	
Nad	◯	◯	◯	◯	◯	Importantísimo

83. Más servicios sociales *

Marca solo un óvalo.

	0	1	2	3	4	
Nad	◯	◯	◯	◯	◯	Importantísimo

84. Mejorar la convivencia *

Marca solo un óvalo.

	0	1	2	3	4	
Nad	◯	◯	◯	◯	◯	Importantísimo

85. Más educación para familias *

Marca solo un óvalo.

	0	1	2	3	4	
Nad	◯	◯	◯	◯	◯	Importantísimo

86. Aumentar la participación social/comunitaria *

Marca solo un óvalo.

	0	1	2	3	4	
Nad	◯	◯	◯	◯	◯	Importantísimo

87. Más servicios sanitarios *

Marca solo un óvalo.

	0	1	2	3	4	
Nad	◯	◯	◯	◯	◯	Importantísimo

88. Mayor compromiso por parte de profesionales que atienden a la infancia *

Marca solo un óvalo.

	0	1	2	3	4	
Nad	◯	◯	◯	◯	◯	Importantísimo

89. Otros. Indique cuáles y añada lo que considere

90. Nombre, COMO MÍNIMO, un par de cosas concretas que se podían aportar desde su centro, recursos, asociación, ámbito para fomentar un barrio más sano (física y emocionalmente) para la infancia y contribuir a acabar con las desigualdades en salud que afectan a niños y niñas de Torrero-La paz

GRACIAS POR SU COLABORACIÓN

El equipo investigador analizará las respuestas y EN SEPTIEMBRE usted recibirá otro cuestionario para llegar al acuerdo entre participantes. Le recordamos que, para que los resultados sean fiables, es muy importante que las personas participantes sean las mismas en todas las fases y que no comparta sus respuestas y reflexiones con el resto de participantes hasta que finalice el proceso de recogida de datos

Este contenido no ha sido creado ni aprobado por Google.

Round 2 Form S1: Questionnaire for the second Delphi round

CUESTIONARIO 2: Delphi activos infancia Torrero

Este estudio forma parte de la Investigación "Desigualdades sociales y de género en salud en la infancia. El papel del capital social y los activos en salud", aprobada por el Comité de Ética en la Investigación de Aragón.

El principal objetivo de esta fase, en la que usted participa, es identificar los activos para la salud (fortalezas y recursos que mejoran y mantienen la salud y que ayudan a acabar con las desigualdades en salud) de la infancia del barrio y las estrategias que podrían fomentarlos.

Como sabe, el equipo investigador debe poder identificar a los y las informantes clave que participan en el Delphi para poder registrar quiénes responden; también se le solicitará el correo electrónico para que, si lo desea, pueda recibir una copia de sus respuestas. Toda la información recogida se tratará conforme a lo establecido en la legislación vigente en materia de protección de datos de carácter personal. En la base de datos del estudio no se incluirán datos personales: ni su nombre, ni ningún dato que le pueda identificar. Se le identificará por un código. Sólo el equipo investigador tendrá acceso a sus datos y nadie ajeno a la investigación podrá consultarlos.
Las conclusiones del estudio se presentarán en congresos y publicaciones científicas, pero se harán siempre con datos agrupados y nunca se divulgará nada que le pueda identificar.

*Es importante que NO comparta sus respuestas e impresiones con otras personas que participan en el Delphi hasta que hayamos finalizado la recogida de respuestas.

PARA CUALQUIER DUDA. INCIDENCIA O CONSULTA PUEDE CONTACTAR CON LA INVESTIGADORA PRINCIPAL:
Isabel Vidal Sánchez.
Mail: vidal@unizar.es
Teléfono: 666914929

* Indica que la pregunta es obligatoria

1. Correo *

____

2. He recibido suficiente información sobre el estudio y comprendo que mi participación es voluntaria: *

Marca solo un óvalo.

- ◯ Sí

Datos participante

3. *Este dato sólo se recoge para asegurarnos de que sólo participan informantes clave. Al analizar los resultados se anonimizarán* NOMBRE: *

____

4. Es usted: *

Marca solo un óvalo.

- ◯ Hombre
- ◯ Mujer
- ◯ Otro: ______________________

5. ÁMBITO EN EL QUE INTERVIENE (marcar sólo uno, el que más represente su labor): *

Marca solo un óvalo.

- ◯ SOCIAL
- ◯ SANITARIO
- ◯ EDUCACIÓN FORMAL
- ◯ EDUCACIÓN NO FORMAL

6. PROFESIÓN:

____

DETERMINANTES DE CRECIMIENTO CRECIMIENTO SANO Y FELIZ

7. Señale el aspecto MÁS IMPORTANTE para la salud y bienestar de la infancia de Torrero-La Paz . Marcar SOLO UNO:

Marca solo un óvalo.

- ◯ Seguridad económica (tener cubiertas las necesidades básicas)
- ◯ Seguridad física (sentirse a salvo)
- ◯ Vivienda digna
- ◯ Estar con su familia
- ◯ Comunicación en la familia fluida y pacífica (que se escuchen y se expliquen las cosas, que no haya gritos..)
- ◯ Tener personas que le quieran mucho
- ◯ Acceso a la educación
- ◯ Hacer actividades que les gusten
- ◯ Acceso a la sanidad
- ◯ Pasar mucho tiempo con la familia
- ◯ Tener talento en algo (música, deporte, arte, tecnología,...)
- ◯ Que su familia le anime en lo que hace bien
- ◯ Disciplina
- ◯ Futuro esperanzador
- ◯ Muchas zonas verdes en el barrio
- ◯ Zonas de juego en el barrio
- ◯ Acceso a la cultura
- ◯ Lugares para la convivencia intercultural (que puedan coincidir, conocerse y relacionarse personas de diversas culturas)
- ◯ Buenas propuestas educativas desde los colegios y talleres escolares
- ◯ Urbanismo integrador
- ◯ Redes comunitarias en el barrio y sentido de comunidad
- ◯ Encontrarse con gente que representa los propios valores la propia comunidad cultural y religiosa.
- ◯ Hacer amigos/as
- ◯ Mejoras en la atención en Servicios Sociales
- ◯ Hacer deporte

8. Añada lo que considere sobre la pregunta anterior

____

9. Señale cuál de estos cuatro aspectos es más deficitario (está peor) para la infancia del barrio (señalar solo uno):

Marca solo un óvalo.

- ◯ Apoyo emocional dentro de la familia
- ◯ Calles
- ◯ Vivienda
- ◯ Futuro esperanzador

10. Señale cuál de estos tres aspectos es más excelente (está mejor) para la infancia del barrio:

Marca solo un óvalo.

- ◯ Redes de apoyo y ayuda mutua
- ◯ Servicios y recursos sanitarios
- ◯ Servicios y recursos educativos
- ◯ Zonas verdes

11. Puede añadir o explicar lo que quiera

____

ACCESO A LA SALUD Y EL BIENESTAR DE LA INFANCIA DE TORRERO

12. ¿Crees que las niñas de Torrero-La Paz nacidas en el seno de una familia española, sin dificultades económicas y que no pertenecen a la etnia gitana tienen la misma posibilidad de acceder a la salud y bienestar que los niños en su misma situación?

Marca solo un óvalo.

- SÍ
- NO

13. ¿Por qué?

14. ¿Quién tiene mayor dificultad para acceder a la salud y el bienestar en el barrio?

Marca solo un óvalo.

- La infancia migrante o cuyos progenitores lo son
- La infancia de etnia gitana

15. ¿Por qué?

Sección sin título

Señale el nivel de dificultad para acceder a la salud y el bienestar de los siguientes niños y niñas:

16. Niñas

Marca solo un óvalo.

EXT 0 1 2 3 4 EXTREMADAMENTE DIFÍCIL

17. Infancia del Pueblo Gitano

Marca solo un óvalo.

EXT 0 1 2 3 4 EXTREMADAMENTE DIFÍCIL

18. Infancia cuya familia procede del Norte de África

Marca solo un óvalo.

EXT 0 1 2 3 4 EXTREMADAMENTE DIFÍCIL

19. Infancia cuya familia procede de África Subsahariana

Marca solo un óvalo.

EXT 0 1 2 3 4 EXTREMADAMENTE DIFÍCIL

20. Infancia cuya familia procede de Latinoamérica

Marca solo un óvalo.

EXT 0 1 2 3 4 EXTREMADAMENTE DIFÍCIL

21. Infancia cuya familia procede de Asia

Marca solo un óvalo.

EXT 0 1 2 3 4 EXTREMADAMENTE DIFÍCIL

22. Infancia cuya familia procede de Rumanía

Marca solo un óvalo.

EXT 0 1 2 3 4 EXTREMADAMENTE DIFÍCIL

23. Niñas y niños con escasos recursos económicos

Marca solo un óvalo.

EXT 0 1 2 3 4 EXTREMADAMENTE DIFÍCIL

24. Niñas y niños con bajo estatus social

Marca solo un óvalo.

EXT 0 1 2 3 4 EXTREMADAMENTE DIFÍCIL

25. Infancia expuesta a la violencia dentro de su ámbito familiar

Marca solo un óvalo.

EXT 0 1 2 3 4 EXTREMADAMENTE DIFÍCIL

26. Infancia expuesta a la violencia fuera de su ámbito familiar

Marca solo un óvalo.

EXT 0 1 2 3 4 EXTREMADAMENTE DIFÍCIL

27. Según su experiencia ¿Cuáles tienen mayor presencia en Torrero-La Paz?

Marca solo un óvalo.

- Niñas
- Infancia del Pueblo Gitano
- Infancia cuya familia procede del Norte de África
- Infancia cuya familia procede de África Subsahariana
- Infancia cuya familia procede de Latinoamérica
- Infancia cuya familia procede de Asia
- Infancia cuya familia procede de Rumanía
- Niñas y niños con escasos recursos económicos
- Niñas y niños con bajo estatus social
- Infancia expuesta a la violencia dentro de su ámbito familiar
- Infancia expuesta a la violencia fuera de su ámbito familiar

28. ¿En qué colectivo habría que centrarse en primer lugar para mejorar el acceso a su salud y bienestar? ¿Con quiénes es más urgente trabajar? Recuerde que en todo momento estamos refiriéndonos a Torrero-La Paz

Marca solo un óvalo.

- ◯ Niñas
- ◯ Infancia del Pueblo Gitano
- ◯ Infancia cuya familia procede del Norte de África
- ◯ Infancia cuya familia procede de África Subsahariana
- ◯ Infancia cuya familia procede de Latinoamérica
- ◯ Infancia cuya familia procede de Asia
- ◯ Infancia cuya familia procede de Rumanía
- ◯ Niñas y niños con escasos recursos económicos
- ◯ Niñas y niños con bajo estatus social
- ◯ Infancia expuesta a la violencia dentro de su ámbito familiar
- ◯ Infancia expuesta a la violencia fuera de su ámbito familiar

29. ¿Por qué?

____

OCUPACIONES/ACTIVIDADES

30. Señale cuál de los siguientes aspectos para que una actividad sea beneficiosa para la salud emocional y mental de las niñas y niños del barrio es el más importante (señalar solo uno):

Marca solo un óvalo.

- ◯ Sentido de competencia (sentir que lo hace bien y/o que va mejorando)
- ◯ Sentimiento de pertenencia
- ◯ Que su familia se sienta orgullosa
- ◯ Aprendizaje
- ◯ Que fomente su autonomía
- ◯ Que le permita desarrollar su creatividad
- ◯ Que fomente su solidaridad y empatía
- ◯ Que sientan que sirve para algo (a nivel personal o de comunidad)
- ◯ Que le ayude a conectar con la capacidad de disfrute
- ◯ Que fomente el desarrollo de su propia identidad
- ◯ Que le permita pasar tiempo de calidad con su familia

31. Añada lo que considere sobre actividades que realiza la infancia del barrio y su relación con la salud y el bienestar

____

PROPUESTAS DE ACCIÓN

32. Para mejorar el barrio para la infancia ¿por dónde empezamos? Señale SOLO UNA opción

Marca solo un óvalo.

- ◯ Presupuesto para infraestructuras
- ◯ Más servicios sociales
- ◯ Mejorar la convivencia
- ◯ Más educación para familias
- ◯ Aumentar la participación social/comunitaria
- ◯ Más servicios sanitarios
- ◯ Más compromiso por parte de las/os profesionales que atienden a la infancia
- ◯ Mayor coordinación y colaboración
- ◯ Colegios activos.
- ◯ Proyectos específicos para familias, proyectos en colaboración con asociaciones de colectivos menos favorecidos para promocionar el ocio y la salud.
- ◯ Entender el bienestar No como tener Más sino como Ser y Sentirse Mejor

33. Puede señalar otras acciones si las considera también prioritarias para mejorar el barrio para la infancia

Selecciona todos los que correspondan.

- ☐ Presupuesto para infraestructuras
- ☐ Más servicios sociales
- ☐ Mejorar la convivencia
- ☐ Más educación para familias
- ☐ Aumentar la participación social/comunitaria
- ☐ Más servicios sanitarios
- ☐ Más compromiso por parte de las/os profesionales que atienden a la infancia
- ☐ Mayor coordinación y colaboración
- ☐ Colegios activos.
- ☐ Proyectos específicos para familias, proyectos en colaboración con asociaciones de colectivos menos favorecidos para promocionar el ocio y la salud.
- ☐ Entender el bienestar No como tener Más sino como Ser y Sentirse Mejor

Office

Sección sin título

A continuación, transcribimos, agrupadas en dos categorías, las propuestas concretas que cada persona participante cree que su centro o recurso podría aportar para mejorar la salud y bienestar de la infancia del barrio:

EDUCACIÓN, CONVIVENCIA, PARTICIPACIÓN Y REDES
Trabajo en valores: tolerancia, respeto, desarrollo de futuros ciudadanos críticos, altruismo, solidaridad, compañerismo, autoconocimiento, amor, etc. Ser un agente activo en la educación no formal y trabajar para acompañar a nuestros educandos a lo largo de su vida.
Mesas de debate, comidas/ meriendas sociales, bailes en comunidad, pasacalles, fiestas de disfraces... cualquier tipo de actividad que diera juego para cohesionar a la comunidad y donde sentirse queridos/as y escuchados/as.
Charlas y talleres de diversos colectivos, como familias diversas, inmigración, refugiados, cafés multiculturales...
La educación como pilar básico de una sociedad sana. Donde se aporte, además de conocimientos (igualdad de oportunidades), desarrollo personal y valores como la solidaridad, la empatía y el respeto mutuo.
Mayor participación de las familias de algunos niños-niñas en la dinámica de los centros escolares para reflexionar en lo importante que es la asistencia a la escuela, que esas familias vivan la escuela como algo suyo.
Trabajar hábitos de higiene y de alimentación.
Red de barrio, trabajo en comunidad.
Mantener una coordinación y un trabajo en red inclusivo con los recursos educativos, de ocio y tiempo libre del barrio.
Educación alimentaria a las familias (incluyendo a madres y abuelas).
Talleres de educación para la salud.
Educación en el colegio con cursos dados por profesionales
Charlas en la escuela a las familias, sobre actividades y propuestas del barrio
Pequeñas formaciones de nuestro idioma para personas migrantes.
Mejorar sistemas de traducción para migrantes incapaces de expresarse en español
Escuela de padres.
Realizar algún tipo de docencia sobre Parentalidad positiva desde el centro de salud o desde educación.
Crear escuelas de padres.
Escuela de padres
Desde el equipo de pediatría se pueden coordinar actividades con los centros educativos.
Además, una correcta educación sanitaria a los niños, les hará adultos comprometidos y responsables con su salud.
Educación en valores y convivencia
Importancia de la salud mental, entendida como concepto amplio
Dinámicas de resolución de conflictos.

ACTIVIDADES, RECURSOS, PROGRAMAS Y AYUDAS
Desarrollar actividades donde la infancia pueda expresarse y que se les tuviera en cuenta desde las administraciones.
Fomentar actividades relacionadas y abiertas para el barrio en las que pueda participar toda la infancia, no sólo la vinculada a nuestro recurso.
Programas de compensación de desigualdades sociales.
Mejorar la accesibilidad a los servicios para todas las familias con necesidades

especiales de logopedia, psicología etc.
Incorporar más recursos o programas específicos orientados al trabajo con la infancia y no solamente con las familias.
Actividades conjuntas con la parroquia para que los niños puedan disfrutar juntos.
Campamentos de verano.
Colaborar con la parroquia en la recogida de alimentos y materiales para las familias del barrio. Ayudas de comedor y de materiales escolares... para todas las familias de nuestro barrio.
Juegos o gymkanas en el parque
Actividades deportivas.
Apertura del patio en más horas y días.
Proyectos educativos en colaboración con el tejido asociativo y comunitario, en relación con el entorno natural (canal, pinares, estepa...), cultural y social.
Actividades de ocio y tiempo libre con mayores recursos y así atender a más.
Ofertar actividades interesantes para adolescentes

34. Comente lo que le sugieren estas propuestas o lo que considere sobre "¿Qué podemos hacer?"

35. PUEDE AÑADIR LO QUE CONSIDERE SOBRE LOS TEMAS TRATADOS, LA INVESTIGACIÓN, LA INFANCIA DEL BARRIO,...:

GRACIAS DE NUEVO POR SU COLABORACIÓN

Si lo desea, el equipo investigador le hará llegar los resultados de la fase en la que ha participado y del estudio completo

36. Quiero conocer los resultados del Delphi en el que he participado *

Marca solo un óvalo.

- SÍ
- NO

37. Quiero conocer los resultados de la investigación completa *

Marca solo un óvalo.

- SÍ
- NO

Este contenido no ha sido creado ni aprobado por Google.

Google Formularios

Table 1 S1: Reasons why Torrero is or is not a good neighbourhood for children to grow up healthy and happy

It is a good neighbourhood for children because	It is NOT a good neighborhood for children because
"Quiet neighbourhood, but with access to everything a city has, more like a village. Green areas nearby. Neighbourhood life".<br>*"Lots of green spaces, squares, parks, and diversity"*<br>*"For its variety of cultures, its parks, its facilities in terms of establishments and its vindictive character, among others".*<br>*"It is a neighbourhood with many services and places that can contribute to the healthy and happy development of the smaller population".*<br>*"Public services, parks, green areas, schools and sports areas, and good communications".*<br>*"It is a neighbourhood that maintains its community and neighbourhood origins, which facilitates the creation of networks, as well as having resources and services aimed at children".*<br>*"Nearby green areas, dynamic neighbourhood with personality".*<br>*"Accessible schools, green areas, local commerce, healthy air".*<br>*"It has many leisure and free time resources, as well as many educational centres, sports activities options and numerous green areas in different parts of the neighbourhood".*<br>*"Growing neighbourhood with increasing child population".*<br>*Good social and community networks. Good natural environment (for a city): Canal, pine forests, steppe. Accessible town planning, although there is room for improvement in many areas, acceptable local commerce".*<br>*"There are various public resources to access, green spaces in the surrounding area, library, etc."*<br>*"Torrero has green areas, a municipal swimming pool, sports centre, shopping centre, etc."*<br>*"It has services for children in many areas, green areas and good communications".*<br>*"It has many social, educational and leisure resources and green spaces to share".*<br>*"The natural environment in which the neighbourhood is located".*<br>*"It is a very family-friendly neighbourhood, with green areas and outdoor spaces for children to play".*<br>*"It is a quiet and family-friendly neighbourhood. It has green areas and streets with little traffic. There are quality public education centres and a variety of organisations that can complement and promote areas outside formal education".*<br>*"Little by little it is getting younger, young families with children are coming here. In addition, it has parks and green areas, a variety of schools, a theatre, a toy library, a library, shops..."*<br>*"It is a small neighbourhood with a lot of variety and places to develop".*	*"Because there are areas in the neighbourhood that do not meet minimum standards of cleanliness. There are areas where the number of conflicts is very high, and children are first line observers. There are areas where families have a very low socio-economic and cultural level and do not have a good understanding of the conditions in which a child should grow up healthy and happy".*<br>*"One part of Torrero la Paz has a low socio-economic and cultural level, depressed and marginalised ethnic and social groups. Urban structure not very favourable: old flats, narrow streets, traffic, etc."*<br>*"It is a neighbourhood with very different areas".*

Table 2 S1: Barriers to children's access to health and well-being

Resources and services and access to them	Physical environment and housing	Economic and social status, cohabitation, belonging, culture	Families: characteristics, habits, styles, parental capabilities, situation
"Excess of population linked to the same health centre, few paediatricians, scarce civic centre, population with social difficulties with which we do not work".<br>"Insufficient paediatricians and nurses for the new demand".<br>"Lack of information about health and child welfare services and resources in the neighbourhood".<br>"School absenteeism"<br>"There is a lack of organisations dedicated to non-formal education work that would allow them to invest their free time and complement formal education".<br>"Lack of information on how to access certain types of aid".<br>"Accessibility".<br>"Access to health care is available to everyone, the difference depends on the professional, on how he/she treats his/her patients".<br>"Lack of investment in the different municipal services"<br>"Lack of education and activities that motivate collective health".<br>"Lack of knowledge of the existing possibilities"<br>"The language".	"Rising rents".<br>"Gentrification".<br>"Poorer housing areas"<br>"Unhealthy housing conditions".<br>"Substandard Housing".<br>"High number of dwellings more than fifty years old that would need some rehabilitation".<br>"In the neighbourhood there is a high number of minors who do not have decent housing".<br>"Unhealthy urban structure (noise, pollution)".	"Lack of economic resources, lack of resources for conflict resolution".<br>"Social isolation".<br>"Risk situation and social and economic exclusion of the family".<br>"1/ Socio-cultural disadvantage; 2/Language problems; 3/Cultural preconceptions; 4/Digital divide".<br>"The families' own situations and the lack of economic resources they have".<br>"Low socio-cultural level of some families, economic and housing difficulties, low expectations for the future".<br>"Situations of risk and social exclusion".<br>"Poor coexistence climate in the neighbourhood".<br>"Sense of belonging to a specific social sector".<br>"Sense of exclusion of a particular social sector".<br>"Economy".<br>"In this neighbourhood there are a large number of families that do not respond to the characteristics indicated as optimal".<br>"There are a large number of children in environments that:<br>- Do not have economic security<br>- They do not see a hopeful future"<br>"Unemployment".<br>"Low socio-cultural level".<br>"Lack of economic security".<br>"Low family economic level"<br>"Low family cultural level"<br>"Aggressive" social environment both in terms of neighbourhood coexistence and peer pressure groups (gangs)".<br>"The economic level of the families"<br>"Cultural level of families"<br>"Poverty"<br>"Prejudices"	"Single-parent families with excessive work schedules or unable to share tasksUnstable family situation".<br>"Unstable family situation".<br>"The need to care for other dependent family members".<br>"Dysfunctional educational styles, poor couple relationships".<br>"Lack of affection in the family"<br>"Practice and incorporation of unhealthy or unhealthy patterns, routines and habits in families, deficiencies or difficulties in education and emotional management during childhood development".<br>"Family problems (destructuring, abandonment, addictions...)".<br>"They are not strengthened when it comes to developing their aptitudes".<br>"Neglect and ignorance".<br>"Family structure".<br>"Lack of availability of caregivers"

Table 3 S1: Improvement proposals for the promotion of children's health in the neighbourhood

Category	Proposals
**Coexistence**	"Strengthen social and community programmes and services (this is not a new idea, it is a commitment)". "More community work"; "cultural, sporting, musical", "intergenerational" and "intercultural" activities and "children's events and workshops that can interest all families living in the neighbourhood regardless of their origin", "courses or school for parents". These should be "from the school so that ALL children can participate, because I believe that the activities outside school hours, those proposed by the neighbourhood, are only attended by a profile of families, not the entire representation of the neighbourhood". "A real network within schools. Avoiding those non-public centres that may segregate them". "To facilitate and create spaces for coexistence". To work with "the associative fabric" and "give prominence to neighbourhood and community networks", strengthening and creating "support networks". "Relationship and coordination of educational, health, social action, etc. areas" and "talks in schools by professionals from other areas". "Improving living conditions (housing, employment... beyond the neighbourhood)”.
**Equality of opportunity:**	Many proposals revolve around strengthening, improving, increasing and caring for "social services" and "public education". They point out that "schools have a fundamental mission in this area, providing the opportunity to mitigate cultural and economic differences", but for them "educational centres must respond to the needs of children and families", "be a support and accompaniment" and "work with the context in your favour and not against it" and for this it would be necessary to guarantee that "the equality plans of educational centres are active documents and that they are really applied and evaluate results".<br>They also propose to guarantee information and access to all sectors of the population and "study and training grants", "free early childhood education",....<br>The participants point out that there are requirements beyond the context of the neighbourhood that have to do with guaranteeing "decent economic resources", "housing" and "employment".
**Accessibility to activities and resources**	They propose "Multidisciplinary programmes to tackle this issue. Study what makes access difficult for each family and draw up an individualised plan for each case, with investment in specialised staff and awareness-raising among the population and professionals in the neighbourhood". Some people propose creating resources, but in general the proposals focus on reinforcing, budgeting for and making accessible the activities and resources that already exist through "information", "free" or "subsidised" activities in which they name sports, leisure and creative activities, advocating "reducing consumerism". "Publicise the leisure time associations that coexist in our neighbourhood and give visibility to organisations such as the PIEES of the educational centres and the extracurricular activities that can be afforded" "Publicise the leisure and educational options that are carried out in the neighbourhood. Inform families in the neighbourhood. Make these activities free of charge". It is also proposed "more community participation" in the design and development of activities, creating "cultural, leisure, solidarity groups, etc.".<br>Above all, two spaces are proposed that can facilitate accessibility: "the school, which is the means of communication that reaches all types of families" and the street through "more street education, more support for free time to be able to take activities to the street and to invite and reach more people". This aspect is also associated with the improvement of the economic situation and the "job market for parents".
**Safety and Autonomy**	The participants advocate improving the physical spaces through "good urbanization and street signage", "better lighting", "pacified roads", "vehicle traffic restrictions", "cleaning of lots and greater vigilance by the authorities" and also for "citizen awareness", "education" and "involving the whole neighbourhood", "promoting solidarity". It is proposed to work "through educational, social and psychological intervention resources" and promote and strengthen projects such as the "Stars Project (school roads)" and work from the "playful", "games" and perform "peer groups to prevent bullying".<br>As a basic pillar to promote the safety and autonomy of children, the family is also pointed out "because this should be worked from home, as a fundamental value" and it also has to do with promoting the solidarity of children towards the elderly, dependents, the sick, etc.
**Spaces and places**	In general, the participants consider that "the places are already there", "they are what they are, "you have to learn to respect them, take care of them and keep them optimal for your enjoyment" and "encourage that what you do" in them is pleasant. "Places exist. There are wonderful ones, but there are no institutions and staff that can help to bring these places closer to all the children in the neighbourhood", "more investment in infrastructure, maintenance" and "cleanliness" to look after and support those that exist "parks and gardens, squares", "CSO Kike Mur", "library", "toy library". "Promote knowledge, appreciation and enjoyment of environments such as the Canal, the Pinares and the Steppe (especially the latter, which is very undervalued)", "Projects in collaboration between schools and the community and associative environment. The same for areas of cultural interest, such as the old prison (today CSO Kike Mur), perhaps the cemetery, points of historical memory...". As proposals for improvement, it is proposed to "improve and condition plots and squares where the people of the neighbourhood (in this case the youngest, but also the oldest) can sit down to share and enjoy", "urban paintings"/"graffiti" in children's areas, "more free leisure and sports areas" "(without capital)".
**Other proposals**	"It is very important to encourage theatre and music groups, literature and cinema for children".<br>"Community policing in conflict areas, with gangs".<br>"Understand wellbeing not as having more but as being and feeling better".<br>"Specific projects for families, projects in collaboration with associations of underprivileged groups to promote leisure and health".<br>"Active schools"
